# Cancer patterns in Arua district, Uganda: a hospital-based retrospective study

**DOI:** 10.3332/ecancer.2024.1688

**Published:** 2024-03-28

**Authors:** Bridget Angucia Sharon, Annet Nakaganda, Geriga Fadhil, Micah June, Ezra Anecho, Gilbert Aniku, Amandua Jacinto, Hesborn Wao, Jackson Orem, Onguru Daniel

**Affiliations:** 1 Uganda Cancer Institute, PO Box 3935, Upper Mulago Hill Road, Kampala, Uganda; 2 Jaramogi Oginga Odinga University of Science and Technology, PO Box 210 - 40601, Bondo, Kenya; 3 Kenya Medical Research Institute (KEMRI), Centre for Global Health Research, PO Box 1578 - 40100, Kisumu, Kenya; 4 Arua Regional Referral Hospital, PO Box 3, Arua, Uganda; 5 New Life Hospice Arua, PO Box 3, Arua, Uganda; 6 African Population and Health Research Centre, PO Box 10787-00100, Kitisuru, Nairobi, Kenya

**Keywords:** population-based cancer registry, Arua district, Uganda, cancer patterns

## Abstract

**Introduction:**

Cancer is the second leading cause of mortality with over 19 million cases and 10 million deaths worldwide. Available data on cancer patterns in Uganda are through modelling of data from two population-based cancer registries (PBCRs) representing only about 10% of the cancer situation in Uganda. This study sought to determine the common types of cancer among adults and children in Arua District over a 5-year period (2017–2021).

**Methods:**

Retrospective cohort chart review and ‘catchment population approach’ were employed. All newly diagnosed cancer patients from Arua between 2017 and 2021 were included in this study. Data were collected using Redcap whereas management and analysis were conducted using Stata 17. Cancer patterns were computed as frequencies and percentages and the interest was in finding out the common cancers among adults (above 19 years) and children (0–19 years).

**Results:**

Over the 5-year study period, a total of 1,118 new cancer cases were registered, with slightly more females (52.1%). The top five common cancers irrespective of sex and age were: liver cancer (13.7%), cervical (11.8%), breast (10.7%), oesophagus (10.5%) and Burkitt’s lymphoma (BL) (6.4%). In this study, 15.3% (*n* = 171) of the study participants were children. The top five common childhood cancers included BL (42%), leukemia (10.5%), other lymphomas (9.4%), osteosarcoma (4.7%) and nephroblastoma (3%).

**Conclusion:**

There is a high incidence of liver cancer in Arua district. The high levels of cervical, breast and oesophagus cancer were consistent with what is reported by the two PBCRs in Uganda. However, BL could be due to the presence of a BL treatment centre at Kuluva hospital in Arua. Cancer interventions in Arua should therefore be targeted towards liver, cervix, breast, and oesophagus cancer and furthering research on the reason for the high incidence of liver cancer.

## Background

Cancer is the second leading cause of mortality worldwide with over 19 million cases worldwide and 10 million deaths [1]. Approximately 50% of all new cancer cases and 70% of all deaths occur in low- and middle-income countries. In Africa, over 1.1 million new cases and 711,429 deaths due to cancer occurred in 2020. Overall, the burden of cancer worldwide is estimated to double by 2030 [2]. In Uganda, 34,008 new cancer cases were registered and 22,992 deaths occurred in 2020. The five common cancers in Uganda are cervix, Kaposi sarcoma (KS), Breast, prostate and non-Hodgkin’s lymphoma [3].

Research has been conducted on the various patterns of cancers in the world, both in developing countries and developed countries. The World Health Organisation, through the International Agency for Research on Cancer (IARC), releases a biannual report of the global cancer statistics (Globocan). These statistics are specific to countries, regions, continents and the world and they describe the patterns of disease, burden and projections of the disease burdens for the next 10 or more years. However, data on cancer patterns in Africa are sparse. The considerable effort in fostering the development of population-based cancer registries (PBCRs) cannot go unnoticed amidst various challenges such as limited human resource, poor medical infrastructure, limited access to diagnostic services, medical and vital records and population denominators. Nonetheless, the information produced by the PBCRs gives a unique insight into cancer patterns in Africa, and is utilised by IARC to estimate and describe the cancer burden for the continent.

Cancer patterns in Uganda is obtained through modelling of data from two PBCRs: Kampala cancer registry (KCR) and Gulu cancer registry (GCR) which covers about 10% of the Uganda population. No periodic surveys are conducted to ascertain the exact magnitude and burden of disease in the different populations yet Uganda is diverse in cultures, ethnicity and environmental exposures which can influence cancer distribution in the country [2]. However, results of a study conducted in Uganda established the feasibility of estimating cancer incidence by employing a retrospective cohort design and ‘catchment population approach’. This approach involves active registration of newly diagnosed cancer cases, for a defined period of time, in all the health facilities serving as a defined population [4]. This study therefore aimed at minimising the gap in knowledge about the cancer burden in Arua District by using a catchment population approach and specifically a population that is not served by either the KCR or the GCR.

This study described the patterns of cancer in Arua district. Specifically, the study sought to determine the common cancers in Arua district over a 5-year period (2017–2021). This would then help strengthen cancer surveillance in Arua which is an important component of cancer control and planning. In addition, the establishment of a regional cancer centre in West Nile sub-region, requires baseline cancer data in the region to inform the planning, implementation and evaluation of cancer control interventions in this region.

## Methods

### Study design and data sources

A retrospective cohort chart review and ‘catchment population approach’ was employed. All newly diagnosed cancer patients from Arua district between 2017 and 2021 were included in this study. Uganda is divided into four regions and ten subregions. West Nile subregion is one of the three subregions in Northern Uganda lying mainly to the west of River Nile. Arua is one of the 12 districts in Westnile subregion. Arua district demarcation, according to the 2014 Uganda Bureau of Statistics census, was used for this study. The population of Arua in the 2014 population census was 782,077 people with about 15% of these as refugees [5]. Uganda has a decentralised health care system comprising of the national referral hospitals; regional hospitals; district hospitals; health centre IV, III, II; and community health workers, commonly known as the village health teams. Services in these public facilities are free of charge. The Private health sector comprises private not for profits, private for profit and traditional and complementary medicine practitioners.

Data were collected using a standardised data abstraction form from health facilities in Arua district. Data collection sites included two hospitals (Kuluva Hospital and Arua Regional Referral Hospital), two health centre IVs, one palliative care centre and Uganda Cancer Institute (the national cancer referral hospital). The variables of interest for this study included sex, age at incidence, incident date, place of residence,

basis of diagnosis, primary site, histological type, treatment received and cancer status at last contact. A cancer case was defined as that diagnosed by clinical, histology of primary tissue, cytology/haematology, specific tumour makers, clinical investigation and histology of metastases. The residence was captured as in the patients’ charts. The address that was written in the charts was what was abstracted and data was abstracted for only cancer patients whose address was Arua. Data quality was ensured by using well-trained research assistants in cancer registration and these were people who had collected data for a similar study carried out in Uganda [4]. A refresher training was done for the research assistants prior to data collection as well as the Human Subject Protection and Good Clinical Practice certification. Ongoing weekly meetings and trainings were held with the research assistants to ensure that standards of cancer registration were not being compromised. Real-time quality control checks of every abstracted form by a trained clinician in cancer registration was done with, differences being resolved on site. Confidentiality was ensured by keeping all study documents under lock and key. Also, the extracted data were kept in a password-protected computer..

### Data analysis

Data entry was collated using REDCap, where validation rules were set to ensure that correct information is entered. This being a hospital-based study and REDCap being readily available to the researchers as an electronic research database for the study was preferred and also the software is more user-friendly and allows for data migration to any other system for continuity. But also because the study had not set out to compute Age Standardised Incidence Rates that required getting baseline population denominators for the different age groups, but rather proportions and frequencies hence preference for REDCap Management was done using REDCap and Stata version 17. Stata version 17 was used to analyse and Excel for data visualisation. Cancer patterns were computed as frequencies and percentages and interest was in finding out the common cancers among adults (both males and females), adult males, adult females and also the common childhood malignancies in Arua district. Proportions of the cancers were obtained and these described the cancer patterns.

## Results

**Figure 1.**
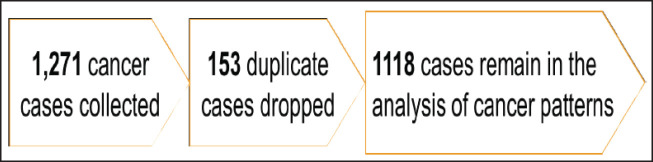
A schema of the cancer cases.

A total of 1,271 cancer cases were collected during the 5-year study period, 153 of these were duplicate cases and therefore dropped from the analysis of the cancer patterns leaving us with 1,118 cases in the final description of cancer patterns of Arua. This is illustrated in Figure 1.

Table 1 is a description of the six sources of data collection for this study, describing their location and what cancer services are offered at these health facilities.

### Characteristics of cancer cases

During the 5-year study period (2017–2021), a total of 1,118 new cancer cases were registered in Arua district with 52.1% females. The median age was 45 years, ranging from 30 to 60 years. The most dominant tribe was the Lugbara (85.2%) who are the largest population in this area. Non-Ugandans accounted for 5.5% of the cancer cases in Arua. Table 2 shows the sociodemographic characteristics of the cancer cases.

### Clinical characteristics of the 1,118 cancer cases in Arua district

Most of the cancer cases were those diagnosed clinically (57.2% of the cases). Seventy-six percent of the cancers were not staged and 88% were alive in their charts at the time of data collection and the date of last contact. About 15% of the cases were from Arua Regional Referral Hospital. Table 3 represents the clinical characteristics of the cancer cases in Arua district.

### Distribution of the cancer cases in Arua district, 2017–2021

**Figure 2.**
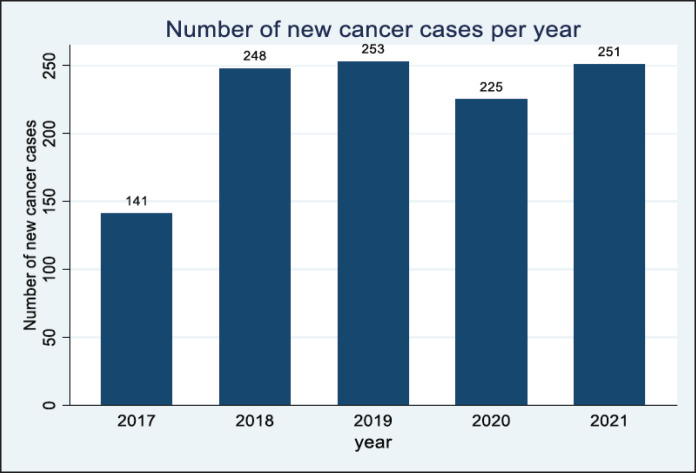
Distribution of the cancer cases in Arua district.

The year 2017 had the least number of cancer cases (X%, *n* = 141 cases) in the 5 years. This was followed by an increase in cases in 2018 and 2019, respectively, however, there was a significant decline in 2020. The year 2021 had an increase in the number of cases from 225 in 2020 to 251 cases in 2021. This is shown in Figure 2.

### Common cancers in Arua district

The commonest cancer seen in Arua district in the 5-year period was liver cancer, 13.7%, irrespective of sex and age. This was followed by cancer of the cervix (11.8%), breast cancer (10.7%), cancer of the esophagus (10.5%) and Burkitt’s lymphoma (BL) (6.4%) Table 4 shows a description of the different cancers in hierarchy of the commonest to the least common in Arua district. The cancer cases were further categorised by age, those aged 20 years and above were categorised as adults while those below 20 years were children and adolescents. These age categories were adopted from the National Cancer Institute which identifies those aged 0–14 years as children, 15–19 years as adolescents and those 20 years and above as adults [6]. Cancer patterns and the common cancers amongst adults and children by sex were then described. About 85% of the cases were adults.

### Common cancers amongst children and adolescents

In this study, 12% (*n* = 138) of the study participants were aged 14 years and below and hence categorised as children. The five most common cancers amongst children from Arua district were BL (49%), other lymphomas (13%), leukaemia (12%), Wilms Tumour (4%) and Retinoblastoma (4%) (Table 5).

### Five common cancers amongst children in Arua, across gender

**Figure 3.**
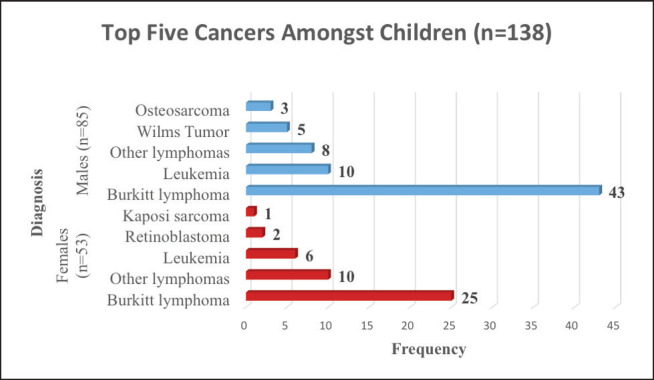
Top five cancers amongst children (age 0–14 years) in Arua.

We found that 61.4% of the children in Arua who had cancer over the 5-year period were males. BL was the commonest cancer amongst both male and female children. As shown in Figure 3, amongst the female children, the commonest cancer was BL (47%), other lymphomas (19%) leukemia (11%), retinoblastoma (4%) and KS (2%). The common cancers amongst male children were; BL (51%), leukemia (12%), other lymphomas (9%), Wilms tumour (6%) and Osteosarcoma (3%) .

### Common cancers amongst adolescents (15–19 years)

About 3% of the study participants were adolescents (aged between 15 and 19 years). The patterns of disease in this age group were characterised by lymphomas mostly accounting for 24%, osteosarcoma 18%, leukemia 15%, BL 12% and KS 9% as illustrated in Table 6.

### Common cancers amongst adults

Adults in this study were those above 19 years and there were 947 cases. The five common cancers amongst adults in Arua district for both sexes were liver cancer (16%), cervix (14%), breast (13%), oesophagus (12%) and KS (5%). Table 7 shows the cancer patterns amongst adults in Arua district.

### Common cancers amongst adult females (n = 519)

Amongst the females, the top five cancers were cervix (25%), breast (22%), liver cancer (13%), lymphomas (4%) and oesophagus (4%).

### Common cancers amongst adult males (n = 428)

**Figure 4.**
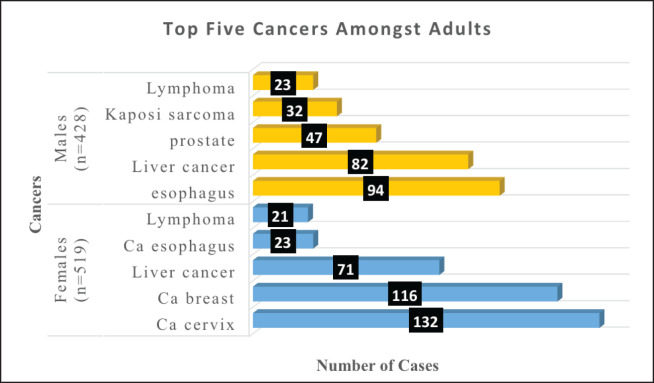
Bar graph of the top five cancers amongst adult females and males in Arua district.

Amongst the males, the top five cancers were esophagus (22%), liver cancer (19%), prostate (11%), KS (7%) and lymphomas (5%). Figure 4 shows the top five cancers amongst adult men and women in Arua district from 2017 to 2021.

## Discussion

The goal of this study was to describe the cancer patterns in Arua district over a 5-year period. The cancer patterns of Arua depicted few numbers in 2017 (141) which could be due to the many missing records in the year 2017. There was a peak in cancer incidence in 2019 (253) and a drop in 2020 (225), the drop in 2020 could be attributed to the lockdown following the COVID19 pandemic in 2020. During the pandemic, the government of Uganda instituted measures to control the spread of the disease including restrictions on movement and a national lockdown from March 2020 to July 2021 [7]. The lockdown was characterised by a ban on public transport but also cancer screening in health facilities hence making it difficult for people to travel to the hospitals for diagnosis [8]. However, cancer incidence increased in the year 2021 (251) and this could be explained by the lift in the lockdown which saw more people accessing health care. Achieving the study goal was dependent on the ability of health facilities in Arua to make a cancer diagnosis [9], clinically or histologically. The ‘catchment population approach’ employed by this study showed that the cancer patterns in Arua are a bit different from those observed in Kampala and Gulu.

The five common cancers in Arua over the 5-year study period were; cancer of the liver, cervix, breast, oesophagus and BL, respectively. The overrepresentation of liver cancer in Arua district could be attributed to the high prevalence of Hepatitis B (HBV) in Uganda with the highest rates in Northern Uganda and Westnile region where Arua district is located. According to 2018 Uganda National Serosurvey, prevalence of HBV was reported to be 4.3% with a 3.8% prevalence in West Nile [10]. HBV infection has been reported as the most common risk factor of liver cancer in Sub-Saharan Africa [11]. However, co-infection with HIV, altered liver function and liver cirrhosis also increase the risk of developing liver cancer [12]. The cancer patterns in Uganda, have never depicted liver cancer to be among the five commonly diagnosed cancers in Uganda. However, liver cancer was the fourth commonest cancer amongst men in Uganda in 2020 accounting for 8.6% of the cancers amongst men in Uganda [1]. However, the role of socioeconomic status as a catalyst for liver cancer risk factors shouldn’t be ignored as this has been evidenced in previous studies [13]. The findings of this study showed that Arua is characterised by low socioeconomic status due to the high levels of unemployment in this district (31.9%). However, the high incidence of liver cancer in Arua could also be attributed to the many people with advanced staged cancer as well as those who were unstaged, this could be an indicator of metastasis to the liver and not necessarily the liver as the primary site of cancer

Cervical cancer was the second commonest form of malignancy overall in Arua and this was different from what Globocan reported in 2020 in Uganda in terms of hierarchy, this is because of the high incidence of liver cancer reported by this study in Arua. There is a possibility that cervical cancer was underestimated in Arua due to cancer diagnostic limitations in the facilities visited. Cervical cancer was reported to be the commonest cancer in Uganda contributing 20.5% of all cancers diagnosed in Uganda [3]. However, cervical cancer was the commonest cancer amongst females in Arua and these findings were consistent with the known cancer trends amongst females in Uganda [14].

Breast cancer, was the third with 10.7%, this was consistent with the national estimates of breast cancer in Uganda. In 2020, breast cancer was the third commonest cancer in Uganda accounting for 7.8% of the cancers in the country. The patterns of breast cancer in Arua were also consistent with what was seen in Uganda in 2018 [15].

Cancer of the esophagus was the commonest cancer amongst men in Arua district and these findings were different from what is reported by IARC. The shift in burden from infection associated to lifestyle-associated cancers in Uganda as shown by a study in Uganda [16] was also seen amongst men in Arua. The district is a tobacco-growing district with a high prevalence of tobacco use, a study done by a student showed that the prevalence of tobacco use among motorcyclists alone in Arua stood at 57% [17] yet, tobacco use and smoking is a known and yet modifiable risk factor for cancer of the oesophagus [18]. In Uganda, cancer of the oesophagus is on the rise hence the high incidence amongst men in Arua.

Prostate cancer was the third commonest cancer amongst men in Aruaat 11%. Worldwide, prostate cancer presents the second common solid tumour amongst men [19]. Although incidence of prostate cancer worldwide has been reported to be on the rise, studies have shown it to stabilise especially in developed countries due to better usage of the prostate-specific antigen for screening and early detection [20]. Prostate cancer was the second commonest cancer in Uganda in 2020 and accounted for 16.3% of the cancers in the country [3].

KS was the fourth common cancer amongst men in Arua at 8.5% of cases. These findings were different from what the previous trends in cancer incidence have shown in Uganda [21]. In 2020, KS was reported as the commonest cancer amongst men in Uganda [22]. However, the underrepresentation of KS amongst men in Arua could be due to the improvement in survival from HIV and the better adherence to ART amongst men in Uganda hence the reduction in progressive HIV [23]. Epidemic KS was reported as the most common type of KS in Uganda and SSA [24]. Therefore, there is a need to maintain the interventions towards the fight against HIV new infections in Uganda and also improve health education on the need for adherence to ART to prevent progression to stage IV HIV at which stage the risk of developing KS is high.

BL was the commonest cancer amongst children accounting for 49% of all cancers in children. The overrepresentation of BL in Arua district could be attributed to the existence of a BL treatment centre in the district specifically at Kuluva Hospital. A PBCR was established at Kuluva in the 1960s, [25], however, this closed down due to inadequate funds. BL was first described in Uganda in 1958 and various research was done by Dr Denis Burkitt to describe epidemiology of the disease but also discoveries for treatment were made by this doctor [26]. Most of this research was done in West Nile and specifically at Kuluva Hospital in Arua where up to date there is still a repository for the early research carried out. The cancer patterns in Uganda have always shown BL to be the most common form of malignancy amongst children, and specifically the endemic BL which is also common in SSA attributed to Epstein Barr virus and *Plasmodium falciparum* malaria [27]. Therefore the high incidence of BL in Arua could be attributed to the endemicity of malaria in Arua [28]. The appearance of BL among the five top cancers in Aruaa could be attributed to the fact that there was a BL diagnosis and treatment centre in Arua district hence the availability of data on BL in the district.

### Strengths and limitations of the study

Ability and willingness of health facilities to provide required information. All the five health facilities visited for data collection provided the required information without any reservations. The ‘catchment population approach’ used correctly described the cancer patterns in the district since the method gives a picture of the cancer burden in Arua. This is the first study in Arua to describe cancer patterns in the district not covered by a PBCRs, these findings will go a long way in designing cancer control interventions in the district. Since the study was based on routine data, it was limited by missing data which could have biased our estimates. Poor record keeping. Some of the health facilities visited had their records already destroyed by natural causes. Archiving methods of old records. Some facilities had no facilities for archiving their old records, because of this, very few records were retrieved for 2017 since these facilities had destroyed some of these records. Pathology laboratories were not visited due to inadequate resources hence reducing the number of morphologically verified cases. Death certificate only cases were also not captured by this study and yet this method is one of the ways of establishing cancer patterns and incidence within the population. Lack of an electronic data management system. Because all the data needed had to be abstracted from the patient files, it made the entire process of data collection cumbersome and time-consuming.

## Recommendations and conclusion

Cancer control planning interventions in Arua should be targeted towards liver cancer by encouraging HBV vaccination and routine testing. The Ministry of health should have targeted Health education to sensitise the masses against HBV.

There should be ongoing screening for breast and cervical cancer in the district by the Ministry of health and partners. The government should plan on decentralising endoscopy services to the people of Arua to ease early detection for cancer of the esophagus as well as creating awareness on cancer of the oesophagus. A government pathology laboratory could also be set up to ease diagnosis.

Establishing of the Arua Cancer Registry and revitalising of the Kuluva cancer registry would go a long way in strengthening cancer surveillance in the region, but also training and employing of more specialists along the cancer control continuum in Arua.

Further research on the high incidence of liver cancer in Arua, specifically a prospective study on the risk factors for liver cancer in the district. However, also increasing the number of morphologically verified liver cancer cases by visiting pathology laboratories.

Cancer surveillance research in resource-limited settings can be done where PBCRs are still scarce. This could then provide detailed and timely information to assess variations in cancer occurrence among different regions of the country and provide a more comprehensive picture of the cancer burden over time, to inform and direct cancer control policies in the country. The cancer patterns described in Arua district can be used for planning cancer control interventions in the West Nile sub-region and subsequently the Arua Cancer registry catchment area. The methods used for this study can be applied to other resource-limited settings, with no PBCRs.

## Conflicts of interest

The author(s) declare that there is no conflict of interest.

## Funding

This study was supported with funding from the Capacity Development of Applied Epidemiologists in Eastern Africa Region (CDAE), a project which is part of the European and Developing Countries Clinical Trials Partnership II (EDCTP2) Programme supported by the European Union. CDAE is jointly implemented by the African Population and Health Research Center (APHRC), the Amref International University (AMIU), Lund University and the Jaramogi Oginga Odinga University of Science and Technology (JOOUST) and funded by the EDCTP2 (grant: CSA2020E139).

## Informed consent

The study obtained a waiver of consent for the participants since the study did not involve direct interaction with the subjects.

## Ethical considerations and institutional review

The study obtained ethical approval from Jaramogi Oginga Odinga University of Science and Technology (JOOUST) Board of Postgraduate studies (Ref;ERC 36/02/23-9/04) and the Makerere University School of Public Health Research and Ethics Committee (Ref;SPH-2022-349). Approval was also obtained from the Uganda National Council of Science and Technology (UNCST) (Ref;HS2539ES). Administrative clearances were obtained from Arua District Local Government, Arua City, Terego District Local Government and Madi Okollo District Local Government for permission to carry out the research in the district.

## Author contributions

Conceptualisation; Angucia Bridget S; Methodology; Angucia Bridget S, Annet Nakaganda and Daniel Onguru; Analysis, Angucia Bridget S; Original draft preparation; Angucia Bridget S; Review and editing; Hesborn Wao, Geriga Fadhil, Micah June, Ezra Anecho, Gilbert Aniku Amandua Jacinto, Jackson Orem and Supervision by Daniel Onguru and Annet Nakaganda.

## Figures and Tables

**Table 1.** Characteristics of the data sources.

Serial #	Name of the health facility	Level of the facility	Ownership	Location	Cancer services offered at the facility
1	Arua Regional Referral Hospital	Regional Referral Hospital	Government	Arua City	Prevention, early detection, diagnosis, treatment, palliative care and surveillance
2	Arua Palliative Care Unit (New Life Hospice)	Hospice	Government	Arua City	Palliative care and surveillance
3	Uganda Cancer Institute	National cancer Referral Hospital	Government	Kampala	Prevention, early detection, diagnosis, treatment, palliative care, research and surveillance
4	Kuluva Hospital	Hospital	Faith based	Arua District	Prevention, early detection, diagnosis, treatment, palliative care and surveillance
5	Omugo HCIV	Health Centre IV	Government	Terego District	Prevention, early detection, diagnosis, palliative care and surveillance
6	RhinoCamp HCIV	Health Centre IV	Government	Madi Okollo district	Prevention, early detection, diagnosis, palliative care and surveillance

This table describes the characteristics of the data sources in terms of their location, ownership and the specific cancer services offered at these health facilities

**Table 2.** Socio-demographic characteristics of the cancer cases in Arua, 2017–2021.

Variable		Frequency	Percent
Sex	Female	583	52.1
	Male	535	47.9
Age category	Adults	947	84.7
	Children	138	12.3
	Adolescents	33	2.9
Tribe	Lugbara	953	85.2
	Madi	55	4.9
	Kakwa	41	3.7
	Alur	25	2.2
	Other	44	3.9
Nationality	Ugandan	1,057	94.5
	South Sudanese	58	5.2
	Congolese	2	0.2
	Ethiopian	1	0.1
Religion	Christian	908	81.2
	Muslim	162	14.5
	Unkown	48	4.3
Marital status	Married	314	28.1
	Single	212	19.0
	Widow/Widower	62	5.6
	Divorced/Separated	50	4.5
	Unknown	480	42.9
Employment status	Unemployed	357	31.9
	Informal employment	245	21.9
	Formal employment	74	6.6
	Unknown	442	39.5

*N* = 1,118, average age was 45 years and the adults were those aged 20 years and above. The dominant tribe were the lugbaras (85.2%) and Christianity was the commonest religion (81.2%)

**Table 3.** Clinical characteristics of 1,118 cancer cases in Arua district.

Variable		Number	Percentage
Basis of diagnosis	Clinical only	639	57.2
	Histology of primary	259	23.2
	Clinical investigations	199	17.8
	Cytology/haematology	15	1.3
	History of metastasis	5	0.5
	Specific tumour markers	1	0.1
Stage	Unstaged	851	76.1
	IV	181	16.2
	III	50	4.5
	II	29	2.6
	I	7	0.6
Status at last contact	Alive	987	88.3
	Dead	131	11.7
Cause of death	This cancer	70	53.4
	Other cause	51	38.9
	Unknown	10	7.63
Place of death	Hospital	81	61.8
	Home	34	25.9
	Unknown	16	12.2
Secondary malignancy	No	1,109	99.2
	Yes	9	0.8
Institution	Arua Regional Referral Hospital	514	43.5
	Arua Palliative Care Unit (New life hospice	268	22.7
	Uganda Cancer Institute	186	15.7
	Kuluva Hospital	196	16.6
	Omugo HCIV	10	0.8
	Rhinocamp HCIV	8	0.7

Most of the cases were not secondary malignancies (99.2%) and 76% of the cancer cases were not staged in the charts while 23.2% were histologically confirmed cases

**Table 4.** New cancer cases in Arua 2017–2021, both sexes, all ages.

ICD-10	Cancer (*n* = 1,118)	Freq	%age
C22-25	Liver cancer	153	13.69
C53	Cervix	132	11.81
C50	Breast	120	10.73
C15	Esophagus	117	10.47
C83.7	BL	72	6.44
C77	Lymphoma	70	6.26
C46	KS	56	5.01
C61	Prostate	47	4.2
C40-41	Osteosarcoma	44	3.94
C42	Leukemia	32	2.86
C16-17	Stomach	24	2.15
C18-20	Colorectal	24	2.15
C56	Ovary	18	1.61
C76.2	Abdominal malignancy	17	1.52
C11	Nasopharyngeal carcinoma	16	1.43
C43	Malignant melanoma	16	1.43
C54	Endometrial cancer	14	1.25
C34	Lung	13	1.16
C01-02	Tongue	11	0.98
C49	Rhabdomyosarcoma	11	0.98
C32	Larynx	11	0.98
C05,C07-09, C30-31	Ear nose and throat cancers	10	0.89
C67	Bladder	9	0.81
C69	Eye	9	0.81
C60	Penis	8	0.72
C64.9	Nephroblastoma	5	0.45
C74	Neuroblastoma	3	0.27
O&U	Other and unspecified cancers	56	5.01

Liver cancer accounted for about 14%, cervix 12%, breast 11%, esophagus 10% and BL 6% of the cancers in Arua district

**Table 5.** Cancer patterns amongst children (Age 0–14 years) in Arua district (*n* = 138).

ICC3 code	Diagnosis	Frequency	Percentage
II	BL	68	49.28
II	Other lymphomas	18	13.04
I	Leukemia	16	11.59
VI	Wilms tumour	5	3.62
V	Retinoblastoma	5	3.62
VIII	Osteosarcoma	4	2.9
IX	KS	3	2.17
IV	Neuroblastoma	3	2.17
XI	Malignant melanoma	2	1.45
IX	Rhabdomyosarcoma	1	0.72
XII	Other unspecified paediatric cancers	13	9.42

*n* = 138 is the number of children with cancer, BL was the commonest cancer 49%, then other lymphomas 13%, leukemia 12% and Wilms tumour 4%

**Table 6.** Cancer patterns amongst adolescents (15–19 years).

ICCC-3	Diagnosis	Frequency	Percentage
II	Lymphoma	8	24.24
VIII	Osteosarcoma	6	18.18
I	Leukemia	5	15.15
II	BL	4	12.12
IX	KS	3	9.09
IX	Rhabdomyosarcoma	1	3.03
XI	Malignant melanoma	1	3.03
XII	Ear nose and throat cancers	1	3.03
XII	Other unspecified adolescent cancers	4	12.5

*n* = 33 is the number of cancers amongst adolescents and the commonest cancer was lymphoma 24%, osteosarcoma 18%, leukemia 15%, BL 12% and KS 9%

**Table 7.** Cancer patterns amongst adults (age >19 years) in Arua, both sexes.

ICD-10	Diagnosis	Frequency	Percentage
C22-25	Liver cancer	153	16.16
C53	Cervix	132	13.94
C50	Breast	120	12.67
C15	Esophagus	117	12.35
C46	KS	50	5.28
C61	Prostate	47	4.96
C77	Lymphoma	44	4.65
C40-41	Bone cancers	34	3.59
C16-17	Stomach	24	2.53
C18-20	Colorectal	24	2.53
C56	Ovary	18	1.9
C76.2	Abdominal malignancy, NOS	17	1.8
C11	Nasopharyngeal carcinoma	16	1.69
C43	Uterus	14	1.48
C54	Lung	13	1.37
C34	Malignant melanoma	13	1.37
C42	Leukemia	11	1.16
C01-02	Tongue	11	1.16
C32	Larynx	11	1.16
C49	Rhabdomyosarcoma	9	0.95
C67	Bladder	9	0.95
C05,C07-09, C30-31	Ear nose and throat cancers	9	0.95
C60	Penis	8	0.84
C69	Eye	4	0.42
O&U	Other unspecified adult cancers	39	4.12

*n* = 947 is the number of cancer cases among adults. Liver cancer 16%, cervix 14%, breast 13%, esophagus 12% and KS 5% were the commonest cancers in Arua
